# 45 Optimising the management of chronic ischemic heart disease by training general practitioners to deliver very brief advice on physical activity (OptiCor)

**DOI:** 10.1093/eurpub/ckae114.020

**Published:** 2024-09-26

**Authors:** Sabrina Kastaun, Sabrina Hoppe, Alicia Prinz, Ute Mons, Daniel Kotz, Andrea Icks, Markus Vomhof, Norbert Donner-Banzhoff, Rik Crutzen, Oliver Kuß, Stefan Wilm

**Affiliations:** Institute of General Practice (ifam), Patient-Physician-Communication Unit, Centre for Health and Society (chs), Medical Faculty and University Hospital of the Heinrich-Heine-University Düsseldorf, Düsseldorf, Germany; Institute of General Practice (ifam), Patient-Physician-Communication Unit, Centre for Health and Society (chs), Medical Faculty and University Hospital of the Heinrich-Heine-University Düsseldorf, Düsseldorf, Germany; Institute of General Practice (ifam), Patient-Physician-Communication Unit, Centre for Health and Society (chs), Medical Faculty and University Hospital of the Heinrich-Heine-University Düsseldorf, Düsseldorf, Germany; Department of Cardiology, Faculty of Medicine and University Hospital Cologne, University of Cologne, Cologne, Germany; Institute of General Practice (ifam), Addiction Research and Clinical Epidemiology Unit, Centre for Health and Society (chs), Medical Faculty and University Hospital of the Heinrich-Heine-University Düsseldorf, Düsseldorf, Germany; Department of Family Medicine, Care and Public Health Research Institute (CAPHRI), Maastricht University, Maastricht, The Netherlands; Institute for Health Services Research and Health Economics, Centre for Health and Society, Medical Faculty and University Hospital Düsseldorf, Heinrich-Heine-University Düsseldorf, Düsseldorf, Germany; Institute for Health Services Research and Health Economics, German Diabetes Center, Leibniz Center for Diabetes Research at Heinrich-Heine-University Düsseldorf, Düsseldorf, Germany; Institute for Health Services Research and Health Economics, Centre for Health and Society, Medical Faculty and University Hospital Düsseldorf, Heinrich-Heine-University Düsseldorf, Düsseldorf, Germany; Institute for Health Services Research and Health Economics, German Diabetes Center, Leibniz Center for Diabetes Research at Heinrich-Heine-University Düsseldorf, Düsseldorf, Germany; Institute of General Practice, Philipps-University Marburg, Marburg, Germany; Department of Health Promotion, Care and Public Health Research Institute (CAPHRI), Maastricht University, Maastricht, The Netherlands; Institute for Biometrics and Epidemiology, German Diabetes Center, Leibniz Center for Diabetes Research at Heinrich-Heine-University Düsseldorf, Düsseldorf, Germany; Institute of General Practice (ifam), Patient-Physician-Communication Unit, Centre for Health and Society (chs), Medical Faculty and University Hospital of the Heinrich-Heine-University Düsseldorf, Düsseldorf, Germany

## Abstract

**Purpose:**

The German treatment guideline “chronic ischemic/coronary heart disease (IHD)” recommends that general practitioners (GPs) deliver advice on physical activity (PA) to IHD patients. This recommendation is inadequately implemented, often due to GP’s insufficient specific training. International guidelines recommend training GPs in how to deliver PA advice effectively and efficiently. Evidence is lacking on how such training should be designed to match the recipients’ needs, and whether it improves the frequency and quality of advice. With OptiCor we aim to systematically develop and evaluate such a training for GPs to optimise the routine care of IHD patients.

**Project:**

OptiCor has started in 2022. It comprises three phases over five years. The methodology matches the Medical Research Council framework recommendations for complex interventions. GPs and patients are substantially involved throughout the entire project. OptiCor (funded by the German Ministry of Education and Research) will be presented internationally for the first time.

Phase 1 (needs analysis/development)–completed: A nationwide, face-to-face household survey was used to collect data on the receipt of GP-delivered PA advice in people with IHD. Focus group discussions and problem-centred interviews were conducted with GPs and patients with IHD to explore attitudes and motivation towards, experiences with, and barriers and facilitators of PA advice implementation or reception, respectively. The findings are now being used to inform the theory-based training development.

Phase 2 (pilot): A pragmatic cluster randomised controlled trial (cRCT) on the effectiveness of the GP training on delivered PA advice during routine care of IHD patients will be pilot tested in summer 2024.

Phase 3 (evaluation): A full pragmatic cRCT will be conducted from 2025 onwards (primary endpoint: patient-reported proportions of GP-delivered PA advice). Health economic and process-related analyses will facilitate a potential future broad dissemination and health economic evaluation of the training.

**Conclusions:**

If our training successfully enhances the proportions and quality of delivered PA advice to IHD patients, it represents a low-threshold strategy for a broad implementation in general practice. The training could extend existing projects on PA referral/prescription schemes, and it could be transferred to the management of other diseases or to other healthcare settings.

